# Loneliness, Social Support, and Psychological Wellbeing Among Older Adults

**DOI:** 10.1093/geroni/igab046.3710

**Published:** 2021-12-17

**Authors:** Poshan Dahal, Eva Kahana, Tirth Bhatta, Polina Ermoshkina

**Affiliations:** 1 Case Western Reserve University, Shaker Heights, Ohio, United States; 2 Case Western Reserve University, Cleveland, Ohio, United States; 3 University of Nevada, Las Vegas, Nevada, United States

## Abstract

Social support in old age has been linked to psychological wellbeing outcomes, such as depressive symptoms. However, insufficient attention has been paid to implications of social support for different domains of psychological wellbeing. In this study, we explored these associations among 797 older adults (mean age = 78.61 years) living in a retirement community in Florida from the ECRC study. Our findings show that measures of social support and connectedness have varying influences on psychological wellbeing. Loneliness was associated with lower life satisfaction (b=- -1.12, p<0.001) and higher depressive symptoms (b=3.52, p<0.001). Higher self-rated social support was associated with higher life satisfaction (b= 1.66, p<0.001) but did not predict depressive symptoms. Depressive symptoms, however, were significantly higher (b=-1.45) among individuals who reported that they don’t have anyone who they can turn to if they feel lonely and want to talk. Feeling lonely also predicted lower positive affects among these older adults (b=-0.65, p<0.001). Similarly, loneliness also predicted higher negative affects (b=1.28, p<0.001). Negative affects were also significantly higher among women (b=-1.15, p<0.001) but lower among those who were living alone (b=-1.06, p<0.001). Overall, our findings underscore the importance of social support and connectedness for psychological wellbeing in later life. This finding is consistent with prior research demonstrating significance of social support in later life for the overall psychological wellbeing of the older adults.

